# Clinical impact of human papillomavirus in laryngeal squamous cell carcinoma: a retrospective study

**DOI:** 10.7717/peerj.3395

**Published:** 2017-05-30

**Authors:** Wei-Chih Chen, Hui-Ching Chuang, Yu-Tsai Lin, Chao-Cheng Huang, Chih-Yen Chien

**Affiliations:** 1 Department of Otolaryngology, Kaohsiung Chang Gung Memorial Hospital and Chang Gung University College of Medicine, Kaohsiung, Taiwan; 2 Head and Neck Oncologic Group, Kaohsiung Chang Gung Memorial Hospital, Kaohsiung, Taiwan; 3 Department of Pathology, Kaohsiung Chang Gung Memorial Hospital and Chang Gung University College of Medicine, Kaohsiung, Taiwan

**Keywords:** HPV, Laryngeal cancer, Survival, Recurrence, Prevalence

## Abstract

**Objectives:**

The purpose of this study is to determine the prevalence and clinical impact of human papillomavirus (HPV) related laryngeal squamous cell carcinoma (LSCC).

**Methods:**

A total of 106 LSCC patients who underwent primary surgical resection with or without adjuvant radiotherapy/chemoradiotherapy were enrolled retrospectively. Tumors collected from paraffin-embedded samples were used for HPV detection by polymerase chain reaction and in situ hybridization technique. Clinicopathological parameters were recorded for analysis.

**Results:**

The prevalence of HPV in patients with LSCC was 13.2% in our series and 12 out of 14 (85.7%) HPV-positive tumors were HPV-16. The patients with HPV-positive tumors were older (*p* = 0.042), less local/regional recurrence (*p* = 0.037) and non-smoker (*p* = 0.068). There was no significant difference in the 5-year overall survival (OS) (*p* = 0.8056) between HPV-positive and -negative tumors. The patients with HPV-positive tumors had a better 5-year disease-specific survival (DSS) (100% *vs*. 84.8%, *p* = 0.1485), although the difference did not reach statistical significance. However, the local/regional control rate was significantly better in HPV-positive tumors than in HPV-negative tumors (100% *vs*. 75%, *p* = 0.0494).

**Conclusions:**

A low prevalence of HPV infection in our series suggests that HPV is not a major cause of LSCC. However, a 100% local/regional control rate and DSS were observed in HPV-positive tumors. This finding suggests a different tumor behavior between HPV-positive and HPV-negative LSCC. Further research with a larger sample size is necessary to confirm our observations.

## Introduction

The high-oncogenic risk types of human papillomavirus (HPV) can induce tumorigenesis via the E6 and E7 viral oncoproteins. These oncoproteins can functionally inactivate the tumor suppressor proteins p53 and pRb, resulting in a loss of cell cycle regulation and immortalization of keratinocytes (Havre et al., 1995; Munger & Howley, 2002). HPV-associated cancers are well documented in cervical cancer, in which 99.7% of cases harbor a high-risk HPV type (Walboomers et al., 1999). At the end of the last century, an association between HPV and head and neck squamous cell carcinoma (HNSCC) was identified, with an overall prevalence of 25% of tumors harboring HPV (Hoffmann et al., 1998; McKaig, Baric & Olshan, 1998; Kreimer et al., 2005). There is mounting evidence of a strong association between HPV and oropharyngeal squamous cell carcinoma (OPSCC), as documented in Europe and the United States (Ang et al., 2010). Currently, HPV-positive and HPV-negative OPSCCs are thought to be two distinct diseases.

Previously, we found that Taiwanese patients with oropharyngeal cancer had a lower prevalence of HPV than that of patients from Western populations (Chien et al., 2008; Armas et al., 2008). In Taiwan, betel nut chewing has a significant impact on health and may cause these differences. Betel nut chewing plays an important role in the development of upper aerodigestive tract malignancies, and synchronous or metachronous tumors of the upper aerodigestive tract are commonly observed in these patients (Su et al., 2013). Lee et al. (2005) also observed that betel nut chewing and tobacco have a synergistic effect on the development of LSCC.

The microenvironment of the laryngeal mucosa is similar to that of the uterine cervix, which has an epithelial junctional area between squamous and columnar epithelia; the junctional area is a potential site for HPV infection (Koskinen et al., 2007). Previous studies have found that low-risk HPV is associated with recurrent respiratory papillomatosis (Bonagura et al., 2010), while high-risk HPV is associated with laryngeal cancer (De Oliveira et al., 2006; Ma et al., 1998; Morshed, 2010). However, the association between laryngeal squamous cell carcinoma (LSCC) and HPV infection remains controversial due to inconsistent results (Lee et al., 2011; Upile et al., 2014; Xu et al., 2014). In a systemic review and meta-analysis, the attributable fractions of HPV infection in LSCC cases were 19.1% and 8.6% according to p16 and E6/E7 mRNA expression, respectively (Ndiaye et al., 2014).

In addition, the relationship between HPV and LSCC has rarely been examined in a primary surgical cohort. Therefore, this study sought to clarify the role of HPV in LSCC and analyzed correlations among HPV, clinicopathological parameters, and clinical outcomes.

## Materials and Methods

### Patients and clinicopathological data

This retrospective study enrolled patients who underwent primary surgical resection with or without adjuvant radiotherapy or chemoradiotherapy between 2006 and 2009 at Chang Gung Memorial Hospital, Kaohsiung, Taiwan. Their clinicopathological characteristics were obtained from clinical records, including age, sex, T and N classification, TNM stage, tumor differentiation, histories of betel nut chewing, alcohol drinking, smoking, and survival. The TNM stage was classified according to the 2009 American Joint Committee on Cancer system as confirmed by the Head and Neck Oncology Group. This study was approved by the Medical Ethics and Human Clinical Trial Committees at Chang Gung Memorial Hospital (Ethical Application Ref: 101-3112B).

### Detection of HPV

Paraffin-embedded samples from identified tumor blocks of each specimen were collected in 1.5-mL Eppendorf tubes for DNA extraction. The tumor blocks were cut after thorough cleaning of the microtome blades, and a blank paraffin section was cut as a control to prevent contamination. After the deparaffinizing procedure, genomic DNA was extracted using the QIAamp tissue kit (Qiagen, Hilden, Germany) according to the manufacturer’s instructions. A total of 50 µL eluted DNA were obtained, of which 1 µL was used as the PCR template. A 192-bp HPV DNA fragment was amplified using MY11/GP6+ biotinylated consensus primers targeting the L1 region of HPV. The DNA integrity of samples was assessed by amplification of β-globin as an internal control. The pre- and post-PCR amplifications were performed in two independent rooms. The HPV-positive samples were all reconfirmed, and HPV-negative samples were randomly selected for repeat procedures to confirm the results. In the HPV-positive samples, HPV was genotyped using a commercial PCR-based reverse-blot assay (EasyChip HPV Blot; King Car, Yilan, Taiwan), which can detect 39 different HPV types. Finally, the HPV types were identified by visual assessment protocol according to the manufacturer’s instructions (Huang et al., 2006; Luo, Roan & Liu, 2007).

### Statistical analysis

Fisher’s exact test was used to evaluate the correlations between clinicopathological variables and HPV status. In all statistical analyses, *p*-values < 0.05 and < 0.1 were considered to indicate significance and marginal significance, respectively. Variables considered in the survival analysis included age, sex, T classification, N classification, TNM stage, tumor differentiation, tumor subsite, second primary cancer, adjuvant therapy, extranodal extension, and the presence of HPV in tumor cells. The Kaplan–Meier method was used for the survival analysis, and statistical significance was defined as *p* < 0.05, as assessed using the log rank test.

## Results

A total of 106 patients (103 men, 3 women; mean age 61.1 ± 11.8 years) were enrolled in this study. Tumor subsites included supraglottic (*n* = 40), glottic (*n* = 54), transglottic (*n* = 11), and subglottic (*n* = 1) cancers. Table 1 summarizes the clinicopathological characteristics. The incidence of a second primary cancer (synchronous or metachronous) was 17.0% (*n* = 18), including esophageal carcinoma in six (5.7%) patients, lung cancer in four (3.8%), other head and neck cancer subsites in six (5.7%), thyroid cancer in one (0.9%), and leukemia in one (0.9%). The second primary head and neck cancers were oral cancer in three, tonsillar cancer in two, and soft palate cancer in one patient.

**Table 1:** Table of clinicopathological features in different HPV status. Correlation between the clinicopathological features and HPV status.

Variables	No.	HPV (−)	HPV (+)	*p* value
Gender				
Male	103	89	14	1.000
Female	3	3	0	
Age				
<60 y/o	52	49	3	0.042^*^
≥60 y/o	54	43	11	
T classification				
T1 and T2	74	64	10	1.000
T3 and T4a	32	28	4	
N classification				
Positive	28	25	3	0.756
Negative	78	67	11	
TNM stage				
Stage I and II	62	54	8	1.000
Stage III and IV	44	38	6	
Extranodal extension				
Positive	16	15	1	0.560
Negative	12	10	2	
Adjuvant RT/CCRT				
Yes	27	24	3	1.000
No	79	68	11	
Tumor recurrence				
Yes	23	23	0	0.037^*^
No	83	69	14	
Second primary cancer				
Yes	18	15	3	0.703
No	88	77	11	
Tumor differentiation				
Well	28	25	3	0.756
Moderate and poor	78	67	11	
Tumor subsites				
Transglottic	11	11	0	0.117
Glottic	54	47	7	
Supraglottic	40	34	6	
Subglottic	1	0	1	
Tobacco use				
Smoking	93	83	10	0.068^**^
Non-smoking	13	9	4	
Alcohol use				
Drinking	51	47	4	0.154
Non-drinking	55	45	10	
Betel nut chewing				
Chewing	53	46	7	1.000
Non-chewing	53	46	7	

**Notes.**

RT radiotherapy CCRT concurrent chemoradiotherapy

* Statistical significance.

** Marginal significance.

### HPV genotyping

**Figure 1:**
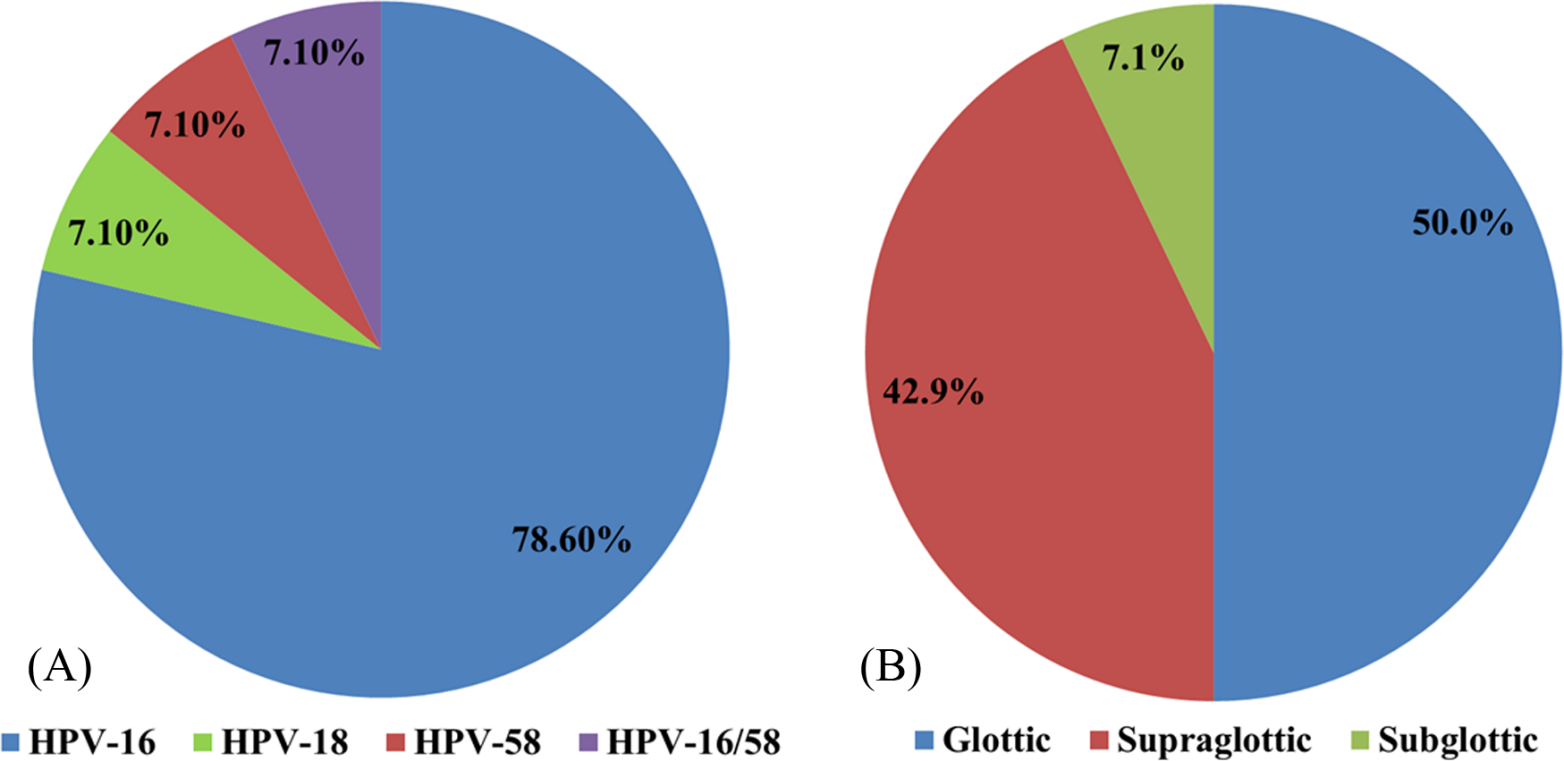
The distributions of different HPV types. The distributions of (A) various HPV types in laryngeal cancer and (B) HPV at different laryngeal subsites.

HPV was detected in 14 patients (13.2%): 11 (78.6%) specimens were positive for HPV-16, one (7.1%) for HPV-18, one (7.1%) for HPV-58, and one (7.1%) for both HPV-16 and -58 (Fig. 1A). HPV was present in the glottic, supraglottic, and subglottic laryngeal subsites in seven (50%), six (42.9%), and one (7.1%) patients, respectively (Fig. 1B).

### Clinicopathological parameters

The patients with HPV-positive tumors were significantly older (*p* = 0.042) and had a higher local/regional control rate (*p* = 0.037) than that of patients with HPV-negative tumors. Fewer patients with HPV-positive tumors were smokers, although this had marginal significance (*p* = 0.068; Table 1).

**Table 2:** Table of outcome according to different clinicopathological features. Actuarial 5-year overall survival, disease-specific survival and local/regional control rate according to the clinicopathological features.

Variable	No	OS	*p*-value	DSS	*p*-value	Recurrence- free	*p*-value
HPV status							
Positive	14	64.3	0.8056	100	0.1485	100	0.0494^*^
Negative	92	67.4		84.8		75.0	
Gender							
Male	103	67.0	0.9186	87.4	0.3010	79.6	0.0490^*^
Female	3	66.7		66.7		33.3	
Age							
<60 y/o	52	76.9	0.0416^*^	86.5	0.9345	78.9	0.8140
≥60 y/o	54	57.4		87.0		77.8	
Tumor differentiation							
Well	28	78.6	0.1416	96.4	0.0818	78.6	0.9210
Moderate/poor	78	62.8		83.3		78.2	
Tumor site							
Glottic	54	81.5	0.0011^*^	96.3	0.0021^*^	81.5	0.2499
Non-Glottic	52	51.9		76.9		75.0	
ENE							
Yes	16	37.5	0.1563	56.3	0.1177	68.8	0.1230
No	12	66.7		83.3		91.7	
T classification							
T1 and T2	74	70.3	0.1762	90.5	0.0589^**^	79.7	0.4137
T3 and T4a	32	59.4		78.1		75.0	
N classification							
Negative	78	73.1	0.0161^*^	93.6	0.0003^*^	78.2	0.9535
Positive	28	50.0		67.9		78.6	
TNM stage							
I, II	62	72.6	0.1044	95.2	0.0021^*^	80.7	0.3744
III, IVa	44	59.1		75.0		75.0	
Second primary cancer							
Yes	18	44.4	0.0338^*^	100	0.1059	88.9	0.3066
No	88	71.6		84.1		76.1	
Adjuvant RT/CCRT							
Yes	27	63.0	0.5087	81.5		88.9	0.1271
No	79	68.4		88.6	0.3121	74.7	

**Notes.**

ENE extranodal extension of lymph node RT Radiotherapy CCRT Concurrent chemoradiotherapy

* Statistical significance.

** Marginal significance.

### Survival analysis

**Figure 2:**
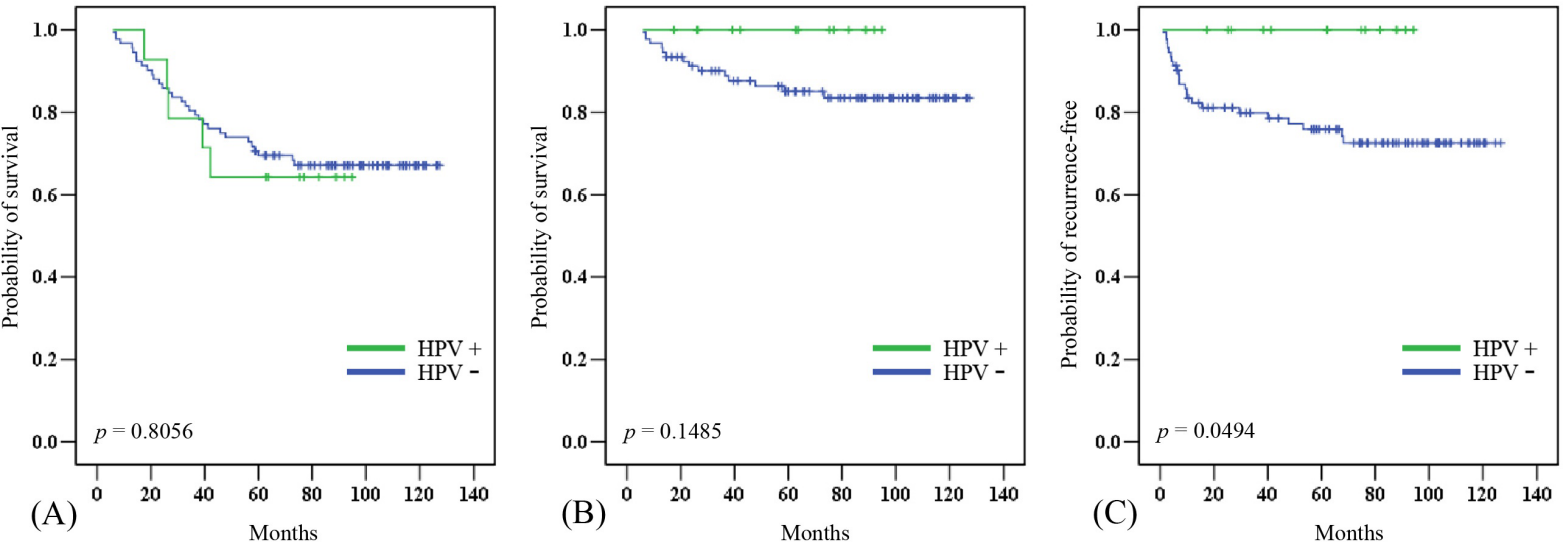
Clinical outcome in HPV-positive and HPV-negative tumors. The effect of HPV status on (A) overall survival, (B) disease-specific survival, and (C) local/regional control rates.

In this cohort, the 5-year overall (OS) and disease-specific (DSS) survival rates were 67.0% and 86.8%, respectively. The median follow-up period was 82.7 (range 6.0–127.4) months. Twenty-one patients died of diseases other than laryngeal cancer, in most cases chronic obstructive pulmonary disease (COPD), lung cancer, or esophageal cancer. The 5-year OS was significantly poorer in patients who were older (*p* = 0.0416), had a positive N classification (*p* = 0.0161), had a second primary cancer (*p* = 0.0338), and had non-glottic cancer (*p* = 0.0011). The 5-year DSS was significantly poorer in patients with a positive N classification (*p* = 0.0003), advanced TNM stage (*p* = 0.0021), and non-glottic cancer (*p* = 0.0021) (Table 2). There was no significant difference in the 5-year OS between HPV-positive and -negative tumors (Fig. 2A). The patients with HPV-positive tumors had a better 5-year DSS (100% *vs*. 84.8%, *p* = 0.1485), although the difference did not reach statistical significance (Fig. 2B). However, the 5-year local/regional control rate was significantly better in HPV- positive tumors than in HPV-negative tumors (100% *vs*. 75%, *p* = 0.0494; Fig. 2C).

## Discussion

Smoking and drinking are the main risk factors for head and neck cancer. Recently, HPV has been shown to be a new pivotal factor in the development of HNSCC, specifically in OPSCC (Stenmark et al., 2017; Wang et al., 2017). However, the role of HPV in head and neck cancers other than OPSCC remains unclear. In oral squamous cell carcinoma (OSCC), although Zafereo et al. (2016) found a high incidence of p16 overexpression (especially in the oral tongue area, 36.3%), only 6% of OSCC cases were considered HPV-driven tumors. In LSCC, the exact role of HPV infection remains controversial, and the prevalence varies widely from 6.8% to 58.8%, with an average prevalence of 28% reported in a recent meta-analysis (Ma et al., 1998; Fouret et al., 1997; Li et al., 2013).

Several factors may contribute to the high variation in HPV prevalence. Lindeberg & Krogdahl (1999) suggested that a higher prevalence could be explained by a high frequency of false-positive results caused by sample contamination. With the technical advances in HPV detection, the prevalence of HPV in patients with LSCC has been quite low in recent publications. In two recent studies involving a large patient series from China and the UK, the prevalence was only 7.57% and 3.2%, respectively (Upile et al., 2014; Xu et al., 2014). Another international cross-sectional study of 3,680 head and neck cancer samples found a low HPV prevalence, except in patients with OPSCC, and only 3.5% of laryngeal cancers were HPV-positive (Castellsagué et al., 2016). We also found a relatively low HPV prevalence: 13.2% of patients with LSCC in Taiwan.

Geographic differences may also contribute to the wide range of HPV infection rates in LSCC. Unlike other countries, betel quid chewing is an important threat to public health in Taiwan in addition to smoking and drinking. Patients who habitually chew betel nut were found to have a higher incidence of supraglottic cancer versus glottic cancer (52.8% *vs*. 35.8%, *p* = 0.003). This suggested that betel nut chewing is a risk factor for the development of supraglottic cancer, in addition to smoking and drinking.

In our study, HPV-16 was the major HPV type, as in other reports (Kreimer et al., 2005). No low-risk HPV types were detected in patients with LSCC in our cohort. Unlike the epidemiological signature of patients with HPV-positive OPSCC, younger patients and those with early T stage disease with extensive nodal metastasis showed no consistent characteristic findings in HPV-positive LSCC (Mallen-St Clair et al., 2016). Xu et al. (2014) found that HPV-positive tumors were associated with supraglottic cancer, non-smokers, and non-drinkers. Hernandez et al. (2014) found a higher prevalence of HPV-positive tumors in women and in patients with node-positive cancer or metastasis. Gillison et al. (2000) found that HPV-positive tumors were more likely to be poorly differentiated. However, we did not find any association of HPV status with clinical stage, nodal metastasis, secondary aerodigestive cancer, tumor subsites, or tumor differentiation (Table 1). In our series, patients with HPV-positive tumors were significantly older and marginally significantly non-smokers (*p* = 0.068). This result is compatible with those of Xu et al. (2014) and Baumann et al. (2009).

Only a few studies have reported the prognosis of HPV-positive LSCC, and these studies failed to show an improved OS (Xu et al., 2014; Hernandez et al., 2014; Shaughnessy et al., 2014). In our series, the OS may not actually reflect the survival advantage of HPV-positive LSCC tumors, since only 14 of 35 deaths were attributed to LSCC. One-third of the deaths were caused by lung cancer, COPD, or esophageal cancer. In addition, none of the patients with HPV-positive LSCC experienced treatment failure, showing 100% local/regional control. HPV-positive LSCC showed a trend toward a better 5-year DSS (100% *vs*. 84.8%, *p* = 0.1485) and a significant improvement in the local/regional control rate (100% *vs*. 75%, *p* = 0.0494). Less aggressive tumor behavior and a better response to adjuvant radiotherapy/concurrent chemoradiotherapy of HPV-positive tumors were possible causes of these clinical outcomes. Shaughnessy et al. (2014) observed an improvement in 2-year DFS in HPV-positive laryngeal and hypopharyngeal cancer patients treated with chemoradiotherapy, but they did not specify the results for LSCC. Although previous reports have not observed a survival advantage in HPV-positive LSCC, we found that patients with HPV-positive LSCC had 100% 5-DSS and 100% local/regional control rates if they underwent primary surgery.

To the best of our knowledge, this is the first report on HPV prevalence in LSCC in Taiwan, where habitual betel nut chewing is common. A limitation of this study is that the presence of HPV DNA in LSCC does not provide sufficient evidence for HPV-induced carcinogenesis. Further biomarkers including p16 and E6/E7 mRNA should be investigated to determine oncogenic activity. However, p16 overexpression is not as reliable of a marker for LSCC as for OPSCC, since it may be triggered by other pathways (Ndiaye et al., 2014). For determination of HPV-induced LSCC in clinical settings, Fusconi et al. (2017) proposed that detection of HPV DNA is the first step, followed by detection of E6/E7 mRNA in positive cases. Currently, limited data on HPV mRNA in LSCC are available, and further prospective studies are required to clarify its roles in prognosis and therapeutic efficacy.

## Conclusions

The prevalence of HPV in patients with LSCC was only 13.2% in our series. The low prevalence of HPV infection suggests that HPV is not a major cause of LSCC. In addition to smoking and drinking, betel nut chewing increases the risk of supraglottic cancer. HPV-related tumors had no significant impact on OS, although a 100% local/regional control rate and 100% 5-year DSS were observed in the patients who underwent primary surgery to treat LSCC. However, a larger sample size is necessary to confirm our observations.

##  Supplemental Information

10.7717/peerj.3395/supp-1 Supplemental Information 1 Raw data

## References

[ref-1] Ang KK, Harris J, Wheeler R, Weber R, Rosenthal DI, Nguyen-Tan PF, Westra WH, Chung CH, Jordan RC, Lu C, Kim H, Axelrod R, Silverman CC, Redmond KP, Gillison ML (2010). Human papillomavirus and survival of patients with oropharyngeal cancer. The New England Journal of Medicine.

[ref-2] Armas GL, Su CY, Huang CC, Fang FM, Chen CM, Chien CY (2008). The impact of virus in N3 node dissection for head and neck cancer. European Archives of Otorhinolaryngology.

[ref-3] Baumann JL, Cohen S, Evjen AN, Law JH, Vadivelu S, Attia A, Schindler JS, Chung CH, Wirth PS, Meijer CJ, Snijders PJ, Yarbrough WG, Slebos RJ (2009). Human papillomavirus in early laryngeal carcinoma. The Laryngoscope.

[ref-4] Bonagura VR, Hatam LJ, Rosenthal DW, De Voti JA, Lam F, Steinberg BM, Abramson AL (2010). Recurrent respiratory papillomatosis: a complex defect in immune responsiveness to human papillomavirus-6 and -11. Acta Pathologica, Microbiologica, et Immunologica Scandinavica.

[ref-5] Castellsagué X, Alemany L, Quer M, Halec G, Quiros B, Tous S, Clavero O, Alos L, Biegner T, Szafarowski T, Alejo M, Holzinger D, Cadena E, Claros E, Hall G, Laco J, Poljak M, Benevolo M, Kasamatsu E, Mehanna H, Ndiaye C, Guimerà N, Lloveras B, León X, Ruiz-Cabezas JC, Alvarado-Cabrero I, Kang CS, Oh JK, Garcia-Rojo M, Iljazovic E, Ajayi OF, Duarte F, Nessa A, Tinoco L, Duran-Padilla MA, Pirog EC, Viarheichyk H, Morales H, Costes V, Félix A, Germar MJ, Mena M, Ruacan A, Jain A, Mehrotra R, Goodman MT, Lombardi LE, Ferrera A, Malami S, Albanesi EI, Dabed P, Molina C, López-Revilla R, Mandys V, González ME, Velasco J, Bravo IG, Quint W, Pawlita M, Muñoz N, De Sanjosé S, Xavier Bosch F (2016). HPV involvement in head and neck cancers: comprehensive assessment of biomarkers in 3680 patients. Journal of the National Cancer Institute.

[ref-6] Chien CY, Su CY, Fang FM, Huang HY, Chuang HC, Chen CM, Huang CC (2008). Lower prevalence but favorable survival for human papillomavirus-related squamous cell carcinoma of tonsil in Taiwan. Oral Oncology.

[ref-7] De Oliveira DE, Bacchi MM, Macarenco RS, Tagliarini JV, Cordeiro RC, Bacchi CE (2006). Human papillomavirus and Epstein-Barr virus infection, p53 expression, and cellular proliferation in laryngeal carcinoma. American Journal of Clinical Pathology.

[ref-8] Fouret P, Monceaux G, Temam S, Lacourreye L, Guily JL (1997). Human papillomavirus in head and neck squamous cell carcinomas in nonsmokers. Archives of Otolaryngology–Head & Neck Surgery.

[ref-9] Fusconi M, Campo F, Gallo A, Zambetti G, Martellucci S, Seccia A, De Vincentiis M (2017). Laryngeal cancer, HPV DNA vs E6/E7 mRNA test: a systematic review. Journal of Voice: Official Journal of the Voice Foundation.

[ref-10] Gillison ML, Koch WM, Capone RB, Spafford M, Westra WH, Wu L, Zahurak ML, Daniel RW, Viglione M, Symer DE, Shah KV, Sidransky D (2000). Evidence for a causal association between human papillomavirus and a subset of head and neck cancers. Journal of the National Cancer Institute.

[ref-11] Havre PA, Yuan J, Hedrick L, Cho KR, Glazer PM (1995). p53 inactivation by HPV16 E6 results in increased mutagenesis in human cells. Cancer Research.

[ref-12] Hernandez BY, Goodman MT, Lynch CF, Cozen W, Unger ER, Steinau M, Thompson T, Saber MS, Altekruse SF, Lyu C, Saraiya M (2014). Human papillomavirus prevalence in invasive laryngeal cancer in the United States. PLOS ONE.

[ref-13] Hoffmann M, Kahn T, Mahnke CG, Goeroegh T, Lippert BM, Werner JA (1998). Prevalence of human papillomavirus in squamous cell carcinoma of the head and neck determined by polymerase chain reaction and Southern blot hybridization: proposal for optimized diagnostic requirements. Acta Oto-Laryngologica.

[ref-14] Huang SL, Chao A, Hsueh S, Chao FY, Huang CC, Yang JE, Lin CY, Yan CC, Chou HH, Huang KG, Huang HJ, Wu TI, Tseng MJ, Qiu JT, Lin CT, Chang TC, Lai CH (2006). Comparison between the hybrid capture II test and an SPF1/GP6+ PCR-based assay for detection of human papillomavirus DNA in cervical swab samples. Journal of Clinical Microbiology.

[ref-15] Koskinen WJ, Brondbo K, Mellin Dahlstrand H, Luostarinen T, Hakulinen T, Leivo I, Molijn A, Quint WG, Roysland T, Munck-Wikland E, Mäkitie AA, Pyykkö I, Dillner J, Vaheri A, Aaltonen LM (2007). Alcohol, smoking and human papillomavirus in laryngeal carcinoma: a Nordic prospective multicenter study. Journal of Cancer Research and Clinical Oncology.

[ref-16] Kreimer AR, Clifford GM, Boyle P, Franceschi S (2005). Human papillomavirus types in head and neck squamous cell carcinomas worldwide: a systematic review. Cancer Epidemiology, Biomarkers & Prevention.

[ref-17] Lee SY, Cho NH, Choi EC, Kim WS, Kim SH (2011). Is human papillomavirus a causative factor of glottic cancer?. Journal of Voice.

[ref-18] Lee KW, Kuo WR, Tsai SM, Wu DC, Wang WM, Fang FM, Chiang FY, Ho KY, Wang LF, Tai CF, Kao EL, Chou SH, Lee CH, Chai CY, Ko YC (2005). Different impact from betel quid, alcohol and cigarette: risk factors for pharyngeal and laryngeal cancer. International Journal of Cancer.

[ref-19] Li X, Gao L, Li H, Gao J, Yang Y, Zhou F, Gao C, Li M, Jin Q (2013). Human papillomavirus infection and laryngeal cancer risk: a systematic review and meta-analysis. The Journal of Infectious Diseases.

[ref-20] Lindeberg H, Krogdahl A (1999). Laryngeal cancer and human papillomavirus: HPV is absent in the majority of laryngeal carcinomas. Cancer Letters.

[ref-21] Luo CW, Roan CH, Liu CJ (2007). Human papillomaviruses in oral squamous cell carcinoma and pre-cancerous lesions detected by PCR-based gene-chip array. International Journal of Oral and Maxillofacial Surgery.

[ref-22] Ma XL, Ueno K, Pan ZM, Hi SZ, Ohyama M, Eizuru Y (1998). Human papillomavirus DNA sequences and p53 over-expression in laryngeal squamous cell carcinomas in Northeast China. Journal of Medical Virology.

[ref-23] Mallen-St Clair J, Alani M, Wang MB, Srivastan ES (2016). Human papillomavirus in oropharyngeal cancer: the changing face of a disease. Biochimica et Biophysica Acta.

[ref-24] McKaig RG, Baric RS, Olshan AF (1998). Human papillomavirus and head and neck cancer: epidemiology and molecular biology. Head & Neck.

[ref-25] Morshed K (2010). Association between human papillomavirus infection and laryngeal squamous cell carcinoma. Journal of Medical Virology.

[ref-26] Munger K, Howley PM (2002). Human papillomavirus immortalization and transformation functions. Virus Research.

[ref-27] Ndiaye C, Mena M, Alemany L, Arbyn M, Castellsagué X, Laporte L, Bosch FX, De Sanjosé S, Trottier H (2014). HPV DNA, E6/E7 mRNA, and p16INK4a detection in head and neck cancers: a systematic review and meta-analysis. The Lancet Oncology.

[ref-28] Shaughnessy JN, Farghaly H, Wilson L, Redman R, Potts K, Bumpous J, Silverman C, Dunlap NE (2014). HPV: a factor in organ preservation for locally advanced larynx and hypopharynx cancer?. American Journal of Otolaryngology.

[ref-29] Stenmark MH, Shumway D, Guo C, Vainshtein J, Mierzwa M, Jagsi R, Griggs JJ, Banerjee M (2017). Influence of human papillomavirus on the clinical presentation of oropharyngeal carcinoma in the United States. The Laryngoscope.

[ref-30] Su YY, Chen WC, Chuang HC, Guo CS, Lin YT, Luo SD, Fang FM, Chien CY (2013). Effect of routine esophageal screening in patients with head and neck cancer. JAMA.

[ref-31] Upile NS, Shaw RJ, Jones TM, Goodyear P, Liloglou T, Risk JM, Boyd MT, Sheard J, Sloan P, Robinson M, Schache AG (2014). Squamous cell carcinoma of the head and neck outside the oropharynx is rarely human papillomavirus related. The Laryngoscope.

[ref-32] Walboomers JM, Jacobs MV, Manos MM, Bosch FX, Kummer JA, Shah KV, Snijders PJ, Peto J, Meijer CJ, Munoz N (1999). Human papillomavirus is a necessary cause of invasive cervical cancer worldwide. The Journal of Pathology.

[ref-33] Wang F, Zhang H, Xue Y, Wen J, Zhou J, Yang X, Wei J (2017). A systematic investigation of the association between HPV and the clinicopathological parameters and prognosis of oral and oropharyngeal squamous cell carcinomas. Cancer Medicine.

[ref-34] Xu Y, Liu S, Yi H, Wang J, Dong P, Li X, Yin S (2014). Human papillomavirus infection in 674 Chinese patients with laryngeal squamous cell carcinoma. PLOS ONE.

[ref-35] Zafereo ME, Xu L, Dahlstrom KR, Viamonte CA, El-Naggar AK, Wei Q, Li G, Sturgis EM (2016). Squamous cell carcinoma of the oral cavity often overexpresses p16 but is rarely driven by human papillomavirus. Oral Oncology.

